# Ritonavir Blocks Hepatitis E Virus Internalization and Clears Hepatitis E Virus In Vitro with Ribavirin

**DOI:** 10.3390/v14112440

**Published:** 2022-11-03

**Authors:** Putu Prathiwi Primadharsini, Shigeo Nagashima, Masaharu Takahashi, Kazumoto Murata, Hiroaki Okamoto

**Affiliations:** Division of Virology, Department of Infection and Immunity, Jichi Medical University School of Medicine, Tochigi 329-0498, Japan

**Keywords:** hepatitis E virus, ritonavir, virus internalization, ribavirin, drug combination, in vitro, virus growth

## Abstract

Hepatitis E virus (HEV) is increasingly recognized as the leading cause of acute hepatitis. Although HEV infections are mostly self-limiting, a chronic course can develop especially in those with immunocompromised state. Ribavirin is currently used to treat such patients. According to various reports on chronic HEV infections, a sustained virological response (SVR) was achieved in approximately 80% of patients receiving ribavirin monotherapy. To increase the SVR rate, drug combination might be a viable strategy, which we attempted in the current study. Ritonavir was identified in our previous drug screening while searching for candidate novel anti-HEV drugs. It demonstrated potent inhibition of HEV growth in cultured cells. In the present study, ritonavir blocked HEV internalization as shown through time-of-addition and immunofluorescence assays. Its combination with ribavirin significantly increased the efficiency of inhibiting HEV growth compared to that shown by ribavirin monotherapy, even in PLC/PRF/5 cells with robust HEV production, and resulted in viral clearance. Similar efficiency was seen for HEV genotypes 3 and 4, the main causes of chronic infection. The present findings provide insight concerning the advantage of combination therapy using drugs blocking different steps in the HEV life cycle (internalization and RNA replication) as a potential novel treatment strategy for chronic hepatitis E.

## 1. Introduction

Hepatitis E virus (HEV) is a quasi-enveloped virus with a single-stranded, positive-sense RNA genome of approximately 7.2 kb and is a member of the family *Hepeviridae,* subfamily *Hepevirinae*, and genus *Paslahepevirus* [1]. The HEV genome possesses the 5′-untranslated region (UTR) capped at its 5′-end, and a short 3′-UTR terminated by a poly(A) tract [2,3]. The genome contains three major open reading frames (ORFs): ORF1, which encodes a non-structural polyprotein involved in viral replication [4,5]; ORF2, which encodes a capsid protein essential for virion assembly and for virion attachment to host cells, and which is the major target for neutralizing antibodies; and ORF3, which is important for virion egress [6,7,8] and is a functional ion channel acting as a viroporin [9].

HEV has two distinct particle forms: the membrane-associated form (eHEV), which is present in the blood stream and culture supernatants, and the membrane-unassociated form (neHEV), which is present in the bile and feces [10,11,12,13].

HEV is distributed worldwide and can be transmitted through various routes, mainly through the fecal–oral and zoonotic foodborne routes [14], as well as through less-frequent routes such as organ transplantation [15], transfusion of blood or blood products [16,17,18,19], and vertical transmission from the mother to the fetus [20]. Despite the low overall mortality rate in the general population, HEV infection can be fatal in pregnant women, where it has shown a 30% mortality rate, particularly in the third trimester of pregnancy [20,21].

There are approximately 20 million HEV infections worldwide every year, and hepatitis E was estimated to cause around 44,000 deaths in 2015 [22]. Although HEV infections are mostly self-limiting, a chronic course can develop, especially in those in an immunocompromised state [15]. Most human infections are with the species *Paslahepevirus balayani* genotypes 1, 2, 3, and 4, and less frequently 7 [1]. Among the four major HEV genotypes infecting humans, a clear epidemiological dichotomy is observed between developing and industrialized countries. HEV genotype 1 (HEV-1) and HEV-2 are restricted to humans and mostly affect people living in Asia and Africa. They are transmitted through fecally-contaminated drinking water [23]. In contrast, HEV-3 and HEV-4 are common in industrialized nations and are transmitted through consumption of raw or undercooked animal meat products [24], organ transplantation [15], or blood transfusion [16,25]. Of note, although HEV-1 and HEV-2 strains usually lead to self-limited infections, HEV-3 and HEV-4 strains can evolve to chronicity in immunocompromised patients [26].

Chronic hepatitis E cases require treatment with antiviral drugs and ribavirin is currently used in such clinical settings [27]. A sustained virological response (SVR)—an undetectable serum HEV RNA level for at least six months after cessation of ribavirin therapy—was achieved in approximately 80% of patients receiving ribavirin monotherapy [28,29,30]. To increase the SVR rate, combination drug treatment might be a viable strategy. 

We recently reported that ritonavir was identified in our screening of a Food and Drug Administration (FDA)-approved library using membrane-associated infectious HEV harboring the nanoKAZ gene in the hypervariable region of ORF1 (eHEV-nanoKAZ [31]). Demonstrating potent inhibition of HEV growth in cultured cells, ritonavir was shown to block early steps in the HEV life cycle such as attachment and internalization [31]. 

In the present study, we showed that ritonavir blocks HEV internalization and that, in cultured cells, its combination with ribavirin exhibited more efficient inhibition of virus growth than ribavirin treatment alone, resulting in viral clearance, even in the PLC/PRF/5 cells with robust production of HEV-3 or HEV-4, which are the main causes of chronic HEV infection [26]. The combination of two drugs blocking different steps in the HEV life cycle—ritonavir as the inhibitor of HEV internalization and ribavirin as the inhibitor of HEV RNA replication—might prove an advantageous novel strategy for treating HEV infection. 

## 2. Materials and Methods

### 2.1. Cell Culture

PLC/PRF/5 (CRL-8024) cells obtained from the American Type Culture Collection (ATCC, Manassas, VA, USA) were grown in Dulbecco’s modified Eagle medium (DMEM) (Thermo Fisher Scientific, Waltham, MA, USA) containing 10% heat-inactivated fetal bovine serum (FBS) (Thermo Fisher Scientific). The growth medium for HEV-infected cells was supplemented with 1% dimethyl sulfoxide (DMSO) (Fujifilm Wako, Osaka, Japan). In the present study, cell culture was conducted under a humidified 5% CO_2_ atmosphere, according to the previously described method [32].

### 2.2. Viruses

Culture supernatants containing the cell-culture-adapted genotype 3 JE03-1760F strain (passage 26; 1.5 × 10^8^ copies/mL) [33] or genotype 4 HE-JF5/15F strain (passage 24; 2.0 × 10^8^ copies/mL) [34,35] were used for virus inoculation (referred to as eHEV-3 [membrane-associated HEV-3] and eHEV-4 [membrane-associated HEV-4], respectively) in this study.

Culture supernatants containing the membrane-associated infectious HEV harboring the nanoKAZ gene in the hypervariable region of ORF1 (eHEV-nanoKAZ [31]) (7.13 × 10^7^ copies/mL) derived from RNA transfection were used for virus inoculation in the luciferase assays.

### 2.3. Drugs

Sucrose (Fujifilm Wako), ribavirin (Fujifilm Wako), and ritonavir (Tokyo Chemical Industry, Tokyo, Japan) were purchased from the indicated sources. 

### 2.4. Quantification of HEV RNA

Total RNA was extracted from culture supernatants using TRIzol-LS reagent (Thermo Fisher Scientific) or from PLC/PRF/5 cells using TRIzol reagent (Thermo Fisher Scientific). The quantification of HEV RNA was performed by real-time reverse transcription (RT)-polymerase chain reaction (PCR) using a LightCycler apparatus (Roche Diagnostics KK, Tokyo, Japan) with a QuantiTect Probe RT-PCR kit (Qiagen, Tokyo, Japan), a primer set, and a probe targeting the overlapping region of ORF2 and ORF3, according to the previously described method [36].

### 2.5. Luciferase Assay

eHEV-nanoKAZ was inoculated into monolayers of PLC/PRF/5 cells in a 96-well plate (Thermo Fisher Scientific) and incubated at 37 °C for 4 h in the presence of the indicated drug dose in DMSO (final concentration, 1%). After inoculation, growth medium containing the indicated drug dose in DMSO (final concentration, 1%) was added to each well, and the cells were subsequently incubated at 35.5 °C for 96 h. The cells were then washed twice with phosphate-buffered saline (pH 7.5) without Ca^2+^ and Mg^2+^ (PBS[–]) and lysed with 20 µL of cell lysis buffer (JNC Corporation, Tokyo, Japan). The intracellular luciferase activity was measured using h-coelenterazine (h-CTZ) (JNC Corporation) in assay buffer for CTZ-type luciferase (JNC Corporation), according to a previously described method [31], in a TriStar^2^ LB942 multimode plate reader (Berthold Technologies, Bad Wildbad, Germany). 

### 2.6. Time-of-Addition Assay

PLC/PRF/5 cells were seeded in a 96-well plate and incubated at 37 °C for 72 h to obtain a monolayer. In the viral inactivation assay, eHEV-nanoKAZ (3 × 10^6^ copies/well) was incubated with ritonavir (20 µM), sucrose (250 mM), or ribavirin (40 µM) with DMSO (final concentration, 1%) in a test tube for 1 h at 37 °C. After washing the monolayers of PLC/PRF/5 cells with PBS(-) twice, the mixture was inoculated into the cells and incubated at 4 °C for 1 h to allow for virus attachment to the cells before being shifted to 37 °C for 1 h to trigger viral internalization. After washing the cells with PBS(-) once to remove any unbound virus, growth medium supplemented with 1% DMSO was added, and the cells were incubated at 35.5 °C. 

In the viral attachment assay, after the monolayers of PLC/PRF/5 cells were washed with PBS(-) twice, eHEV-nanoKAZ was inoculated into the cells along with the indicated drug in DMSO (final concentration, 1%). Following incubation at 4 °C for 1 h, the treated cells were incubated at 37 °C for 1 h. After washing the cells with PBS(-) once, growth medium supplemented with 1% DMSO was added, and the cells were then incubated at 35.5 °C.

In the viral internalization assay, the monolayers of PLC/PRF/5 cells were washed with PBS(-) twice and then inoculated with eHEV-nanoKAZ and incubated at 4 °C for 1 h. After removal of the inoculum, the indicated drug in DMSO (final concentration, 1%) was added and the cells were then incubated at 37 °C for 2 h. After washing the cells with PBS(-) once, growth medium supplemented with 1% DMSO was added, and the cells were then incubated at 35.5 °C.

In all experiments, after 96-h incubation at 35.5 °C, the cell lysates were collected, and then the intracellular luciferase activity was measured, as previously described [31]. 

eHEV-nanoKAZ-infected PLC/PRF/5 cells treated with drug vehicle—DMSO (final concentration, 1%)—were used as the control for each assay.

### 2.7. Immunofluorescence Assay

In a four-well chamber slide (Watson, Tokyo, Japan), monolayers of PLC/PRF/5 cells were inoculated with eHEV-3 at 2 × 10^5^ copies/well in the presence of ritonavir (20 µM), sucrose (250 mM), or ribavirin (40 µM) and incubated at 37 °C for 4 h. After washing the cells with PBS(-) twice, the growth medium supplemented with 1% DMSO was added, and the cells were incubated at 35.5 °C for 96 h. The cells were then subjected to immunofluorescence staining according to the previously described method [37]. The primary antibody used was anti-HEV ORF2 monoclonal antibody (MAb, H6225) [36], and the secondary antibody was Alexa-Fluor 488-conjugated anti-mouse IgG (Thermo Fisher Scientific). Nuclei were counterstained with 4′,6-diamidino-2-phenylindole dihydrochloride (DAPI; Thermo Fisher Scientific). Slide glasses were mounted with Fluoromount/Plus medium (Diagnostic BioSystems, Pleasanton, CA, USA) and then viewed under an FV1000 confocal laser microscope (Olympus, Tokyo, Japan).

### 2.8. Validation of the Anti-HEV Activity of the Ritonavir and Ribavirin Combination Therapy

eHEV-nanoKAZ was inoculated into PLC/PRF/5 cells in a 96-well plate in the presence of 49 drug combination doses: 0, 0.05, 0.1, 0.5, 1, 5, and 35 µM for ritonavir and 0, 1, 5, 10, 20, 40, and 80 µM for ribavirin. Each dose of ritonavir and ribavirin applied in this experiment was determined according to the doses used in our previous report [31]. The cells were then incubated at 37 °C for 4 h. Growth medium containing the indicated drug dose in DMSO (final concentration, 1%) was then added to each well, and followed by incubation at 35.5 °C for 96 h. The cell lysates were then collected, and the intracellular luciferase activity was measured according to the previously described method [31]. 

### 2.9. Cell Viability Assay

Cell viability was measured using a Cell Counting kit-8 (Dojindo Laboratories, Kumamoto, Japan) according to the manufacturer’s protocol. In brief, PLC/PRF/5 cells were seeded in a 96-well plate and incubated at 37 °C for 48 h. The indicated doses of drugs in DMSO (final concentration, 1%) were then added to each well, followed by the incubation of the cells at 37 °C for 96 h. Subsequently, highly water-soluble tetrazolium salt (WST-8) solution was added to each well, and the cells were incubated at 37 °C for 2 h. Absorbance was measured at 450 nm using an iMark microplate reader (Bio-Rad Laboratories, Hercules, CA, USA). Measured values were normalized to the value of the DMSO (vehicle) control.

### 2.10. Evaluation of the Efficacy of Ritonavir and Ribavirin Combination Therapy in a Cell Culture System 

Monolayers of PLC/PRF/5 cells in a 24-well plate (Thermo Fisher Scientific) were inoculated with 5 × 10^4^ copies of eHEV-3 or eHEV-4 in growth medium without FBS, containing the indicated drug dose in DMSO (final concentration, 1%), and then subsequently incubated at 37 °C for 2 h. After incubation, the cells were washed five times with PBS(-), and 0.5 mL of growth medium containing the indicated drug dose in DMSO (final concentration, 1%) was added to each well, followed by incubation at 35.5 °C. Every other day, half of the culture medium was replaced with fresh growth medium containing the indicated drug dose in DMSO (final concentration, 1%). The collected culture supernatants were centrifuged at 1300× *g* at room temperature for 2 min, and the supernatants were stored at −80 °C until use. The cell lysates from the final observation day (48 dpi) were collected and subjected to real-time RT-PCR to quantitate the intracellular HEV RNA. The dose of ritonavir and ribavirin applied in this experiment was determined according to our previous report [31] and the results of the validation of the anti-HEV activity of the drug combination in the current study. The dose of ribavirin was 40 µM, while those of ritonavir were 5, 10, 20, and 35 µM. 

### 2.11. Evaluation of the Efficacy of Ritonavir and Ribavirin Combination Therapy Using PLC/PRF/5 Cells That Robustly Produce HEV 

The eHEV-3 or eHEV-4 was inoculated into monolayers of PLC/PRF/5 cells. After the HEV RNA titer in culture supernatants of the infected cells reached the plateau stage, they were collected. A mixture of naïve PLC/PRF/5 cells at 1 × 10^5^ cells/well and 1 × 10^2^ HEV-infected cells/well was prepared and seeded into a 24-well plate and then incubated at 35.5 °C. After 48 h, the culture supernatants were removed, the cells were washed with PBS(-) five times, and 0.5 mL of growth medium containing the indicated drug dose in DMSO (final concentration, 1%) was added to each well. The cells were incubated at 35.5 °C. Every other day, half of the culture medium was replaced with fresh growth medium containing the indicated drug dose in DMSO (final concentration, 1%). The collected culture supernatants were centrifuged at 1300× *g* at room temperature for 2 min, and the supernatants were then stored at −80 °C until use. The cell lysates from final observation day (60 days after the start of drug treatment) were collected and subjected to real-time RT-PCR to quantify the intracellular HEV RNA. The dose of ritonavir and ribavirin applied in this experiment was determined according to our previous report [31], and the validation results regarding the anti-HEV activity of the drug combination in the current study. The dose of ribavirin was 40 µM, while those of ritonavir were 5, 10, 20, and 35 µM.

### 2.12. LDH Cytotoxicity Assay

The cytotoxicity of the drug treatment was quantified by measuring lactate dehydrogenase (LDH) activity released into the culture medium using an LDH cytotoxicity assay kit (Nacalai Tesque, Kyoto, Japan) according to the manufacturer’s protocol. In brief, 100 µL culture supernatants in a 96-well plate were added with 100 µL substrate solution. The plate was protected from light and incubated for 20 min at room temperature. Following the addition of 50 µL stop solution, absorbance was measured at 490 nm using an iMark microplate reader. Measured values were normalized to the value of vehicle control.

### 2.13. Calculation of the Degree of Synergism with Ritonavir and Ribavirin Combination Therapy

The synergy of ritonavir and ribavirin combination therapy was quantified by comparing the observed drug combination responses (dose–response matrix) against the expected combination responses, calculated using a reference model that assumes no interaction between the two drugs. The degree of synergism was quantified by synergy scoring models (reference model). The reference model used in this study is the highest single agent (HSA), which states that the expected combination effect is the maximum of the single drug responses at corresponding concentrations (SynergyFinder ver. 2) [38]. The degree of inhibition of intracellular luciferase activity by each combination was determined to calculate and visualize the synergy score, as was that of the dose–response curve and matrix. A total of 49 drug combinations were used. The doses for ritonavir were 0, 0.05, 0.1, 0.5, 1, 5, and 35 µM, while those for ribavirin were 0, 1, 5, 10, 20, 40, and 80 µM. The doses used in this study were based on our previous report [31]. The interaction between the two drugs was classified as antagonistic when the synergy score was <−10, additive when it was between −10 and 10, and synergistic when the score was >10.

### 2.14. Statistical Analyses

The results were presented as the mean ± standard deviation (SD). Statistical significance was assessed by Student’s *t*-test. *p* values of <0.05 were considered statistically significant. 

## 3. Results

### 3.1. Ritonavir Blocks HEV Internalization

**Figure 1:**
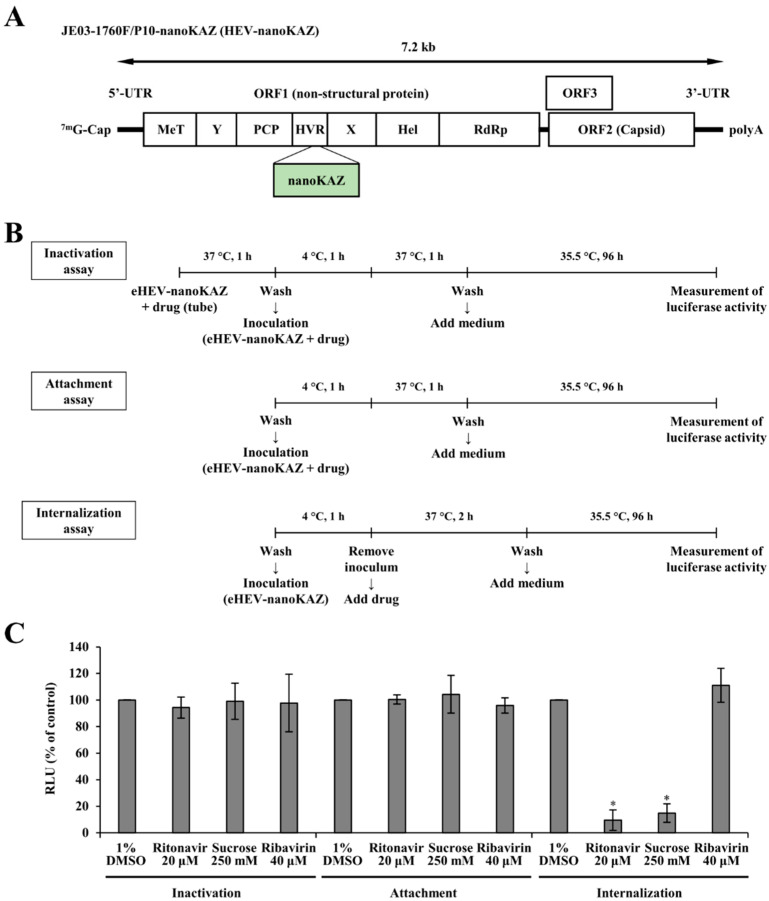
Determination of the steps in the HEV life cycle blocked by ritonavir by a time-of-addition assay. (**A**) A schematic diagram of the infectious HEV harboring the nanoKAZ gene in the hypervariable region of ORF1 (JE03-1760/P10-nanoKAZ: HEV-nanoKAZ). MeT, methyl transferase; Y, Y domain; PCP, papain-like cysteine protease; HVR, hypervariable region; X, macro domain; Hel, helicase; RdRp, RNA-dependent RNA polymerase. (**B**) A schematic representation of the time-of-addition assay that includes an inactivation assay, attachment assay, and internalization assay, using the cell-culture-generated, membrane-associated HEV-nanoKAZ (eHEV-nanoKAZ). (**C**) PLC/PRF/5 cells were inoculated with eHEV-nanoKAZ, with ritonavir (20 µM) introduced at different time points and conditions to represent the viral inactivation, attachment, or internalization. The intracellular luciferase activity was determined four days post-inoculation and was compared to that of the untreated control cells. Sucrose (250 mM) and ribavirin (40 µM) served as reference drugs. The data represent the mean ± standard deviation (SD) of two independent experiments. * *p* < 0.005.

In our previous report, ritonavir was hypothesized to inhibit early steps of the HEV life cycle, such as attachment and internalization [31]. To determine which early stage in the HEV life cycle was blocked by ritonavir, a time-of-addition assay using eHEV-nanoKAZ (Figure 1A) was performed, consisting of viral inactivation, viral attachment, and viral internalization assays (Figure 1B). As references, we utilized sucrose to represent an inhibitor of clathrin-mediated endocytosis [39], as both viral particle forms of HEV (eHEV and neHEV) are known to depend on clathrin-mediated endocytosis [12]; and ribavirin, an inhibitor of HEV RNA replication [27].

The viral inactivation assay was performed to examine whether or not ritonavir inactivates eHEV in a cell-free state and prevents subsequent infection. Treatment with ritonavir, which was pre-incubated with eHEV-nanoKAZ [31] before being inoculated into PLC/PRF/5 cells, did not decrease the intracellular luciferase activity (94.3%) (Figure 1C). Similarly, in the viral attachment assay, ritonavir also did not decrease the intracellular luciferase activity (100.4%) (Figure 1C). In contrast, ritonavir significantly decreased the intracellular luciferase activity to 9.6% (*p* = 0.0036) in the internalization assay, similar to the result shown by treatment with sucrose (14.9%, *p* = 0.0033) (Figure 1C). However, ribavirin did not decrease intracellular luciferase activity in the viral inactivation assay, viral attachment assay, or the viral internalization assay (Figure 1C). 

**Figure 2:**
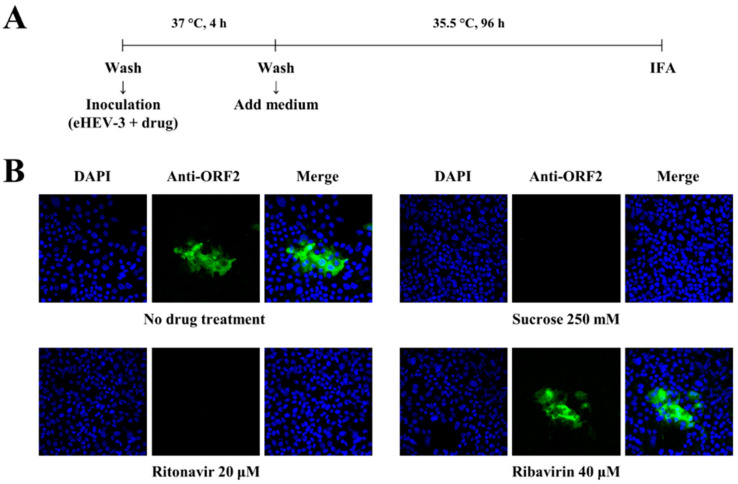
Confirmation of the inhibition of HEV internalization by ritonavir using an immunofluorescence assay. (**A**) A schematic representation of the immunofluorescence assay. (**B**) Immunofluorescence staining of the PLC/PRF/5 cells at day 4 post-inoculation with cell-culture-generated, membrane-associated genotype 3 HEV (JE03-1760F: eHEV-3) to examine the HEV ORF2 protein expression after treatment with ritonavir (20 µM), sucrose (250 mM), or ribavirin (40 µM), and compared to that of the untreated control cells. Results representative of one of two experiments are shown.

To confirm these findings, an immunofluorescence assay (IFA) was performed to examine the expression of ORF2 protein in PLC/PRF/5 cells inoculated with eHEV-3 in the presence of ritonavir, sucrose, or ribavirin (Figure 2A). The ORF2 protein expression was detectable in a control well with no drug treatment and that treated with ribavirin (Figure 2B). The expression of ORF2 protein was undetectable in the cells inoculated with eHEV-3 in the presence of ritonavir, similar to the result shown by treatment with sucrose (Figure 2B). Taken together, these results indicated that ritonavir blocks HEV internalization.

### 3.2. Validation of the Anti-HEV Activity of Ritonavir and Ribavirin Combination Therapy against HEV Using the eHEV-nanoKAZ System

**Figure 3:**
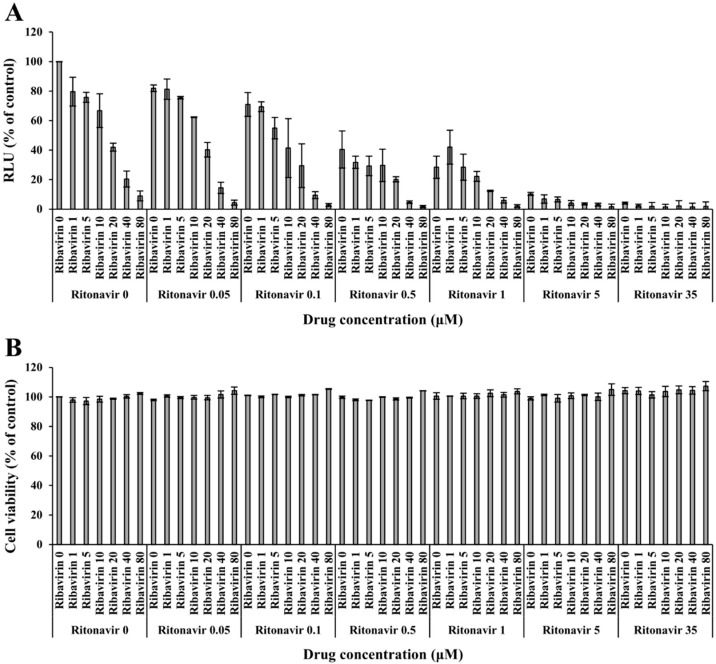
Validation of the anti-HEV activity of the ritonavir and ribavirin combination. (**A**) The intracellular luciferase activity of the eHEV-nanoKAZ-inoculated PLC/PRF/5 cells after treatment with ritonavir and ribavirin combination at various concentrations, in comparison to that of untreated control cells. The cells were lysed four days after the drug treatment. Data represent the mean ± SD of two independent experiments. (**B**) A cell viability assay to examine the effect of the ritonavir and ribavirin combination on cellular proliferation and survival, performed four days after the drug treatment. Data represent the mean ± SD of duplicate wells.

To validate the anti-HEV activity of ritonavir and ribavirin combination therapy, the eHEV-nanoKAZ system was utilized. eHEV-nanoKAZ was inoculated into PLC/PRF/5 cells in the presence of 49 drug combination doses, ranging from 0 to 35 µM for ritonavir and 0 to 80 µM for ribavirin. The drug combination decreased the intracellular luciferase activity in a dose-dependent manner (Figure 3A) without exerting any significant effects on the cellular proliferation or survival, as confirmed by a cell viability assay (Figure 3B).

### 3.3. Evaluation of the Efficacy of Ritonavir and Ribavirin Combination Therapy for Inhibiting Virus Growth in PLC/PRF/5 Cells Inoculated with eHEV-3 or eHEV-4

**Figure 4:**
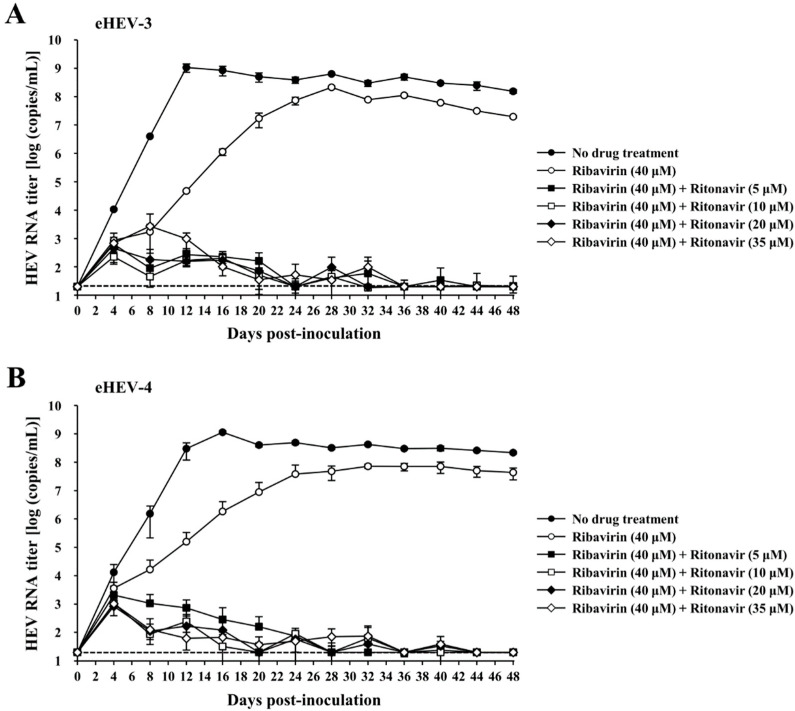
Efficacy of the ritonavir and ribavirin combination against HEV in cultured cells. The HEV growth kinetics were observed for 48 days in PLC/PRF/5 cells inoculated with either cell-culture-generated, membrane-associated genotype 3 HEV (JE03-1760F: eHEV-3) (**A**) or genotype 4 HEV (HE-JF5/15F: eHEV-4) (**B**) in the presence of ribavirin (40 µM) combined with various concentrations of ritonavir, and compared to those of ribavirin monotherapy (40 µM). The data are presented as the mean ± SD of triplicate wells. The dotted horizontal line represents the limit of detection by real-time RT-PCR used in this study, at 2 × 10^1^ RNA copies/mL.

To evaluate the efficacy of the ritonavir and ribavirin combination in cultured cells, eHEV-3 or eHEV-4 was inoculated into PLC/PRF/5 cells in the presence of the indicated drug doses in DMSO (final concentration, 1%). The dose of ritonavir and ribavirin was determined according to our previous report [31], and the results from the validation of the anti-HEV activity of the drug combination in the current study. The efficacy of the combination treatment of ribavirin (40 µM) and various doses of ritonavir (5, 10, 20, and 35 µM) was compared to that of ribavirin monotherapy (40 µM), considering that ribavirin is administered as a single treatment in chronic hepatitis E infection. The eHEV-infected PLC/PRF/5 cells with no drug treatment served as controls. The HEV RNA levels in the culture supernatants were quantified, and the virus growth was observed for 48 days. The HEV RNA titers of the wells with no drug treatment increased and reached 1.5 × 10^8^ copies/mL and 2.2 × 10^8^ copies/mL on day 48 post-inoculation in the eHEV-3- and eHEV-4-inoculated PLC/PRF/5 cells (Figure 4A,B). In both eHEV-3- and eHEV-4-inoculated PLC/PRF/5 cells, treatment with ribavirin monotherapy initially inhibited HEV growth; however, the HEV RNA titers gradually increased and reached 2.0 × 10^7^ copies/mL (Figure 4A) and 4.3 × 10^7^ copies/mL (Figure 4B), respectively, by the final observation day (48 dpi). 

All of the combination treatments exhibited strong inhibition of virus growth in both eHEV-3- and eHEV-4-inoculated PLC/PRF/5 cells, with the HEV RNA titers in culture supernatants becoming undetectable in all wells by the end of the observation period (48 dpi) (Figure 4A,B). The negativity of HEV RNA in the culture supernatants at 48 dpi was consistent with the undetectable intracellular HEV RNA in the corresponding wells (Table 1). The concentration of released LDH into the culture supernatants by the end of the observation period suggested that the drug combination did not cause any significant cytotoxicity (Table 2).

These results indicate that the combination of ritonavir and ribavirin inhibited HEV growth more efficiently than the inhibition exhibited by ribavirin monotherapy in both eHEV-3- and eHEV-4-inoculated PLC/PRF/5 cells. 

### 3.4. Evaluation of the Efficacy of Ritonavir and Ribavirin Combination Therapy for Inhibiting Virus Growth in PLC/PRF/5 Cells Robustly Producing eHEV-3 or eHEV-4

**Figure 5:**
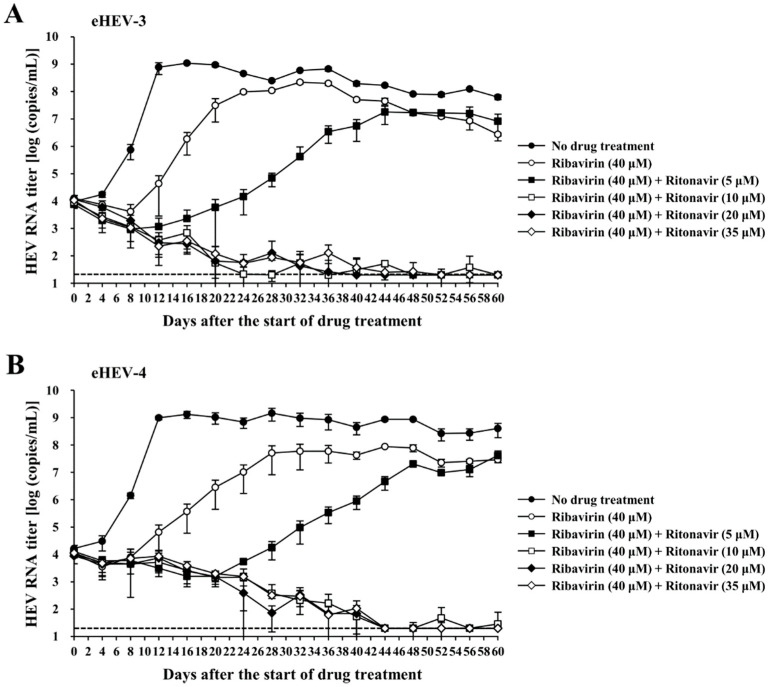
Efficacy of the ritonavir and ribavirin combination against HEV in the culture system consisting of either eHEV-3 (cell-culture-generated, membrane-associated genotype 3 HEV [JE03-1760F])—(**A**) or eHEV-4 (cell-culture-generated, membrane-associated genotype 4 HEV [HE-JF5/15F])—(**B**) producing PLC/PRF/5 cells, in the presence of ribavirin (40 µM) combined with various concentrations of ritonavir, in comparison to that of ribavirin monotherapy (40 µM). HEV growth was observed for 60 days in the presence of various concentrations of the drug combination. The data are presented as the mean ± SD of triplicate wells. The dotted horizontal line represents the limit of detection by real-time RT-PCR used in this study, at 2 × 10^1^ RNA copies/mL.

To model the condition of HEV-infected patients, cells robustly producing eHEV-3 or eHEV-4 (PLC/PRF/5 cells infected with eHEV-3 or eHEV-4 for a long period of time, continuously producing viruses in a high titer) were used to further evaluate the efficacy of ritonavir and ribavirin combination therapy. Various concentrations of the drug combination were applied to the eHEV-3- or eHEV-4-producing PLC/PRF/5 cells. The drug concentration was determined according to our previous report [31], as well as the results from the validation of the anti-HEV activity of the drug combination in the current study. Considering that ribavirin is administered as monotherapy in chronic HEV infection, we included single ribavirin treatment in this evaluation, in order to compare the efficacy of ribavirin and ritonavir combination with that of ribavirin monotherapy. The HEV RNA levels in the culture supernatants on the seeding day was approximately 10^4^ copies/mL in all wells (Figure 5A,B). Observation was performed for 60 days after the start of drug treatment. The HEV RNA titer in the wells with no drug treatment continued to increase and reached 6.3 × 10^7^ copies/mL and 4.0 × 10^8^ copies/mL in the eHEV-3- (Figure 5A) and eHEV-4- (Figure 5B) producing cells by the end of the observation period (60 days after the start of the drug treatment).

Inhibition of virus growth was exhibited in the first week of observation of the cells treated with ribavirin alone (40 µM), in both eHEV-3- and eHEV-4-producing cells. However, it gradually increased and reached 2.7 × 10^6^ copies/mL and 2.9 × 10^7^ copies/mL on day 60 after the start of drug treatment for both eHEV-3- and eHEV-4-producing cells (Figure 5A,B). The combination of ribavirin (40 µM) and ritonavir (5 µM) exhibited more efficient inhibition of HEV growth than ribavirin monotherapy (40 µM), although the HEV RNA titer gradually increased during the observation period (Figure 5A,B). In contrast, the combination of ribavirin (40 µM) and ritonavir (10, 20, and 35 µM) significantly inhibited HEV growth in both eHEV-3- and eHEV-4-producing cells, resulting in undetectable levels of HEV RNA in culture supernatants by the final day of observation (60 days after the start of drug treatment) (Figure 5A,B). The undetectable HEV RNA in the culture supernatants was further supported by the undetectable intracellular HEV RNA by the end of the observation period in all corresponding wells (Table 3). There was no significant cytotoxicity caused by the drug treatment as suggested by the concentration of LDH in the culture supernatants from the final day of cultivation (Table 4). 

Taken together, these results indicated that ritonavir and ribavirin combination therapy effectively inhibited HEV growth, even in a culture system consisting of cells that robustly produce HEV, and the combination exerted similar inhibition for both eHEV-3- and eHEV-4-producing cells.

### 3.5. The Degree of Synergism of Ritonavir and Ribavirin Combination Therapy

**Figure 6:**
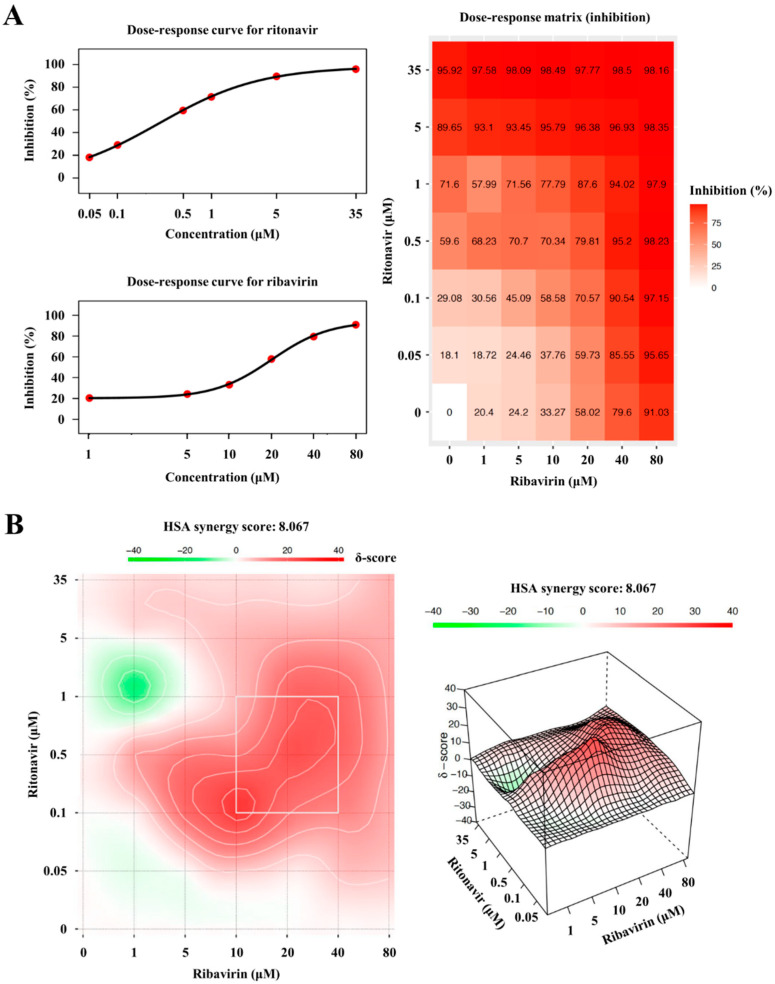
Calculation and visualization of synergy score of the ritonavir and ribavirin combination by SynergyFinder 2.0. (**A**) Dose–response curve for ritonavir (upper left panel), dose–response curve for ribavirin (lower left panel), and dose–response matrix for ritonavir and ribavirin combination (right panel), calculated based on the inhibition level of the intracellular luciferase activity of the eHEV-nanoKAZ (cell-culture-generated, membrane-associated HEV-nanoKAZ)-inoculated PLC/PRF/5 cells treated with indicated drug concentration. The cells were lysed four days after the drug treatment. (**B**) Synergy maps of the drug combination generated by using the highest single agent (HSA) model, visualized in two-dimensional (**left** panel) and three-dimensional (**right** panel) figures. Synergy scores indicate the interactions between the two drugs, where a score of < −10 indicates that the interaction between the two drugs was antagonistic, a score of −10 to 10 indicates that it was additive, and a score of > 10 indicates that it was synergistic.

To determine the synergy of ritonavir and ribavirin combination, the browser-independent web application SynergyFinder version 2.0 was utilized in this study. The inhibition responses from a total of 49 combination doses used in Figure 3A were applied for the calculation. Dose–response plots of phenotypic responses for the single drug (i.e., response measurements, when the other drug doses were 0), fitted by four-parameter logistic curve, are presented for ritonavir (Figure 6A, upper left panel) and ribavirin (Figure 6A, lower left panel). The observed drug combination responses (Figure 6A, right panel) were compared with the expected combination responses calculated by the HSA reference model to determine the degree of synergism of ritonavir and ribavirin combination therapy, yielding a synergy score of 8.067 (the interaction between the two drugs classified as additive), visualized in two- (Figure 6B, left panel) and three-dimensional (Figure 6B, right panel) synergy maps.

## 4. Discussion

Chronic HEV infection predominantly occurs in immunocompromised patients, with a majority of cases reported in organ transplant recipients, while fewer reports involve human immunodeficiency virus (HIV)-infected patients, patients receiving anti-cancer therapy, and patients receiving immunosuppressants [26]. In this setting, ribavirin has been used as an anti-HEV drug [27]. According to various reports on chronic HEV infections, an SVR—an undetectable serum HEV RNA level for at least six months after cessation of ribavirin therapy—was achieved in approximately 80% of patients receiving ribavirin monotherapy [28,29,30]. As chronic HEV infections mainly occur in immunocompromised patients with multiple underlying diseases and morbidities, achieving an SVR and preventing relapse should be the relevant treatment goals, since these will greatly help improve their quality of life in addition to allowing medical professionals to focus on the treatment of their underlying diseases. In view of this, drug combinations might be a viable alternative strategy to increase the SVR achieved by ribavirin monotherapy.

Studies on drug combination for chronic hepatitis E treatment have been reported concerning several drugs, such as sofosbuvir—a nucleotide analog inhibitor of hepatitis C virus (HCV) NS5B polymerase—with some conflicting results [26], or with pegylated interferon-alpha, which can increase the risk of acute rejection in transplant recipients and subsequent graft loss [40]. 

In our previous report involving screening using the eHEV-nanoKAZ system with an FDA-approved drug library to search for potential novel anti-HEV drug candidates, ritonavir was identified. It potently inhibited HEV growth in cultured cells, not only naïve PLC/PRF/5 cells inoculated with eHEV-3 but also in cells with robust production of eHEV-3 [31]. Ritonavir is a protease inhibitor routinely prescribed to HIV-infected patients (lopinavir/ritonavir) [41]. It is exclusively used as a pharmacokinetic enhancer of other protease inhibitors owing to its potent inhibition on the cytochrome P4503A4 (CYP3A4), a major human drug-metabolizing enzyme [42]. The use of ritonavir in other viral diseases has also been reported for the treatment of chronic hepatitis C (ombitasvir/paritaprevir/ritonavir [43,44,45]) and, recently, in the treatment of coronavirus disease 2019 (COVID-19) (nirmatrelvir/ritonavir [46]). Regarding the safety of ritonavir treatment, the Antiretroviral Pregnancy Registry (APR) reports no evidence of an increased risk of human teratogenicity [47]. 

In our recent report, ritonavir was hypothesized to inhibit early steps in the HEV life cycle, such as attachment and internalization [31]. To examine in detail which steps in the HEV life cycle were inhibited by ritonavir, eHEV-nanoKAZ (Figure 1A) was used to perform a time-of-addition assay (Figure 1B). The results suggested that ritonavir blocks HEV internalization (Figure 1C), which was further supported by IFA images where the ORF2 expression was undetectable in the HEV-inoculated cells treated with ritonavir (Figure 2B), thus confirming our hypothesis. Although ritonavir is widely recognized as a protease inhibitor that acts as a booster for other protease inhibitors [42], we showed that even as monotherapy it exerted significant inhibition of HEV growth, particularly in cultured cells [31], which in the current study was demonstrated to be due to its inhibition of HEV internalization. 

We attempted to combine ribavirin with ritonavir in the present study. Target-wise, this is a potential novel strategy for the treatment of chronic HEV infection where ribavirin, an HEV RNA replication inhibitor, was combined with ritonavir, which was found to be blocking HEV internalization in the current study (Figure 1C and Figure 2B). We first examined the inhibition effect of the ritonavir and ribavirin combination on the luciferase activity in the eHEV-nanoKAZ-inoculated PLC/PRF/5 cells in the presence of various doses of the combination. The drug combination decreased the intracellular luciferase activity in a dose-dependent manner (Figure 3A) without causing any significant effect on the cell viability (Figure 3B), indicating that the decreased intracellular luciferase activity was not due to cytotoxicity. 

To examine the efficacy of this combination for long-term treatment, we performed an evaluation in cultured cells. Considering that chronic HEV infection can be caused by HEV-3 and HEV-4, we used both genotypes for the evaluation. In this evaluation, the drug concentration was determined according to our previous report [31] on the evaluation of the efficacy of single ritonavir to inhibit HEV growth in cultured cells, as well as from the results of the validation of the anti-HEV activity of the drug combination in the current study. Since ribavirin monotherapy is currently used as the treatment for chronic HEV infection, we included single ribavirin treatment in this evaluation to compare the efficacy of ritonavir and ribavirin combination against that of ribavirin monotherapy.

In naïve PLC/PRF/5 cells inoculated with either eHEV-3 or eHEV-4, combination of ritonavir and ribavirin exerted more potent inhibition of virus growth than ribavirin monotherapy (Figure 4A,B). The strong inhibition by this drug combination could be seen from the early days of treatment and was maintained throughout the observation period. By the final day of observation (48 dpi), this combination cleared the virus in both culture supernatants (Figure 4A,B) and intracellularly (Table 1) with similar potency in the eHEV-3- and eHEV-4-inoculated cells. 

Furthermore, this efficacy was shown in the PLC/PRF/5 cells with robust production of eHEV-3 or eHEV-4, where the drug combination also demonstrated more efficient inhibition of virus growth than ribavirin monotherapy (Figure 5A,B). The combination of ribavirin (40 µM) and higher concentrations of ritonavir (10, 20, and 35 µM) was able to suppress the production of HEV RNA to undetectable levels in the culture supernatants of both eHEV-3- and eHEV-4-producing PLC/PRF/5 cells (Figure 5A,B) as well as intracellularly (Table 3), further supporting the potential use of this combination as an alternative strategy for the treatment of chronic HEV infection. 

Collectively, this combination significantly increased the efficiency of inhibiting HEV growth, compared to that shown by ribavirin monotherapy, achieving an undetectable HEV RNA level in both naïve PLC/PRF/5 cells inoculated with HEV and in PLC/PRF/5 cells with robust HEV production (Figure 4 and Figure 5), without causing any significant cytotoxicity (Table 2 and Table 4). In addition, combining an HEV RNA replication inhibitor with an HEV internalization inhibitor might help prevent viral rebound after the cessation of ribavirin treatment, thus reducing the relapse rate, as has been reported in a study on chronic HCV infection models where the entry inhibitors were demonstrated to limit viral rebound following discontinuation of the direct-acting antiviral (DAA) treatment [48].

Chronic HEV infection requires at least a three-month treatment course, and this can be extended to six months depending on the patient’s clinical condition [27]; therefore, the use of a ritonavir and ribavirin combination, which has been recognized as a part of long-term treatment combinations in HIV infection and chronic HCV infection, is rational. Although ritonavir inhibits CYP3A4 [49], the results of in vitro studies using both human and rat liver microsome preparations indicated little or no cytochrome P-450 enzyme-mediated metabolism of ribavirin, with minimal potential for P-450 enzyme-based drug interactions [50], and therefore, ritonavir does not act as a booster to ribavirin, and the use of this combination is reasonable. However, as ritonavir potently inactivates the major drug-metabolizing enzyme CYP3A4 [49], and patients requiring treatment for chronic HEV infection are mainly immune-suppressed and receiving multiple drugs for their underlying conditions, caution must be taken if this drug combination is to be used in the clinical setting. The degree of synergy with this combination was assessed using the HSA reference model (SynergyFinder ver.2), where the score was 8.067 (Figure 6B), indicating that the interaction between the two drugs was additive. Therefore, the dose of ribavirin may be able to be reduced and thereby mitigate the side effects, particularly anemia [28,29,30], caused by its administration at a high dose as a single treatment in chronic HEV infection.

Although chronic HEV infection in immunocompromised patients has been widely reported, it was rarely detected in HIV-infected patients [26]. Following the first report on an HIV-infected patient suffering from chronic HEV infection [51], where ritonavir was a part of his antiretroviral therapy (ART) regimen [52], additional cases have been rare, compared to the frequent detection of chronic HEV infection in solid organ transplant recipients—1–2% in European countries [26]. A recent report from a cohort in Namibia demonstrated that HIV-infected women, particularly those who received the ART regimen, appeared to be at lower risk to develop fulminant course of HEV infection [53]. There were four different ART regimens including ritonavir in the study, and therefore it may be possible that the inhibition effect by ritonavir might have caused milder disease presentation in chronic HEV infection in the HIV-infected patients receiving the ritonavir-boosted ART regimen. However, we cannot rule out the role of immune mechanism in these circumstances, where the antiretroviral agents may possibly attenuate immune response to mitigate the level of hepatitis [53,54]. These subjects would be interesting to investigate in the future.

In conclusion, we demonstrated that ritonavir blocks HEV internalization in vitro. Compared to ribavirin monotherapy, the combination of ritonavir and ribavirin exhibited more efficient inhibition in both eHEV-3 and eHEV-4 growth in cultured cells. The combination of drugs targeting two distinct steps in the HEV life cycle (ritonavir as an inhibitor of HEV internalization, and ribavirin as an inhibitor of HEV RNA replication) might be a viable novel strategy for hepatitis E treatment in the future, warranting further study in vivo. It would also be interesting to elucidate the role of ritonavir in the less frequent occurrence of chronic HEV infection in HIV-infected patients. In addition, the inclusion of HEV-1 in the evaluation of the efficacy of the ritonavir and ribavirin combination may be considered, as certain acute hepatitis E cases also require antiviral treatment, such as those developing fulminant course, or those at risk of developing such a course (the elderly, patients with underlying liver disease, or those in immunocompromised state).

## Figures and Tables

**Table 1:** Intracellular HEV RNA at the final observation day (48 dpi) in eHEV-3- and eHEV-4-inoculated PLC/PRF/5 cells.

Treatment	Intracellular HEV RNA *	
	eHEV-3	eHEV-4
No drug treatment	5.9 × 10^8^ copies/well (mean)	1.1 × 10^8^ copies/well (mean)
Ribavirin (40 μM)	1.2 × 10^8^ copies/well (mean)	4.4 × 10^7^ copies/well (mean)
Ribavirin (40 μM) + Ritonavir (5 μM)	Undetectable in all wells	Undetectable in all wells
Ribavirin (40 μM) + Ritonavir (10 μM)	Undetectable in all wells	Undetectable in all wells
Ribavirin (40 μM) + Ritonavir (20 μM)	Undetectable in all wells	Undetectable in all wells
Ribavirin (40 μM) + Ritonavir (35 μM)	Undetectable in all wells	Undetectable in all wells

* Data represent the result from triplicate wells.

**Table 2:** Lactate dehydrogenase (LDH) released into the culture supernatants of eHEV-3- and eHEV-4-inoculated PLC/PRF/5 cells at the final observation day (48 dpi).

Treatment	LDH Release (Mean ± SD) *	
	eHEV-3	eHEV-4
No drug treatment	23.1% ± 7.1%	2.7% ± 1.0%
Ribavirin (40 μM)	19.5% ± 5.4%	2.7% ± 1.0%
Ribavirin (40 μM) + Ritonavir (5 μM)	4.6% ± 1.9%	3.4% ± 1.8%
Ribavirin (40 μM) + Ritonavir (10 μM)	1.9% ± 0.3%	1.9% ± 0.3%
Ribavirin (40 μM) + Ritonavir (20 μM)	3.9% ± 0.2%	2.7% ± 1.2%
Ribavirin (40 μM) + Ritonavir (35 μM)	13.0% ± 2.2%	12.8% ± 0.8%

* LDH release was determined in the culture supernatants from the final day of cultivation (48 dpi) to examine the cytotoxicity caused by the drug treatment. Data represent the mean ± SD of triplicate wells.

**Table 3:** Intracellular HEV RNA at the final observation day (60 days after the start of drug treatment) in the culture system consisting of eHEV-3- and eHEV-4-producing PLC/PRF/5 cells.

Treatment	Intracellular HEV RNA *	
	eHEV-3	eHEV-4
No drug treatment	1.4 × 10^8^ copies/well (mean)	2.5 × 10^8^ copies/well (mean)
Ribavirin (40 μM)	1.6 × 10^7^ copies/well (mean)	2.3 × 10^7^ copies/well (mean)
Ribavirin (40 μM) + Ritonavir (5 μM)	2.0 × 10^7^ copies/well (mean)	1.5 × 10^7^ copies/well (mean)
Ribavirin (40 μM) + Ritonavir (10 μM)	Undetectable in all wells	Undetectable in all wells
Ribavirin (40 μM) + Ritonavir (20 μM)	Undetectable in all wells	Undetectable in all wells
Ribavirin (40 μM) + Ritonavir (35 μM)	Undetectable in all wells	Undetectable in all wells

* Data represent the result from triplicate wells.

**Table 4:** Lactate dehydrogenase (LDH) released into the culture supernatants of eHEV-3- and eHEV-4-producing PLC/PRF/5 cells at the final observation day (60 days after the start of drug treatment).

Treatment	LDH Release (Mean ± SD) *	
	eHEV-3	eHEV-4
No drug treatment	15.9% ± 1.5%	8.1% ± 2.4%
Ribavirin (40 μM)	10.9% ± 0.9%	3.2% ± 1.0%
Ribavirin (40 μM) + Ritonavir (5 μM)	4.0% ± 1.4%	3.0% ± 1.8%
Ribavirin (40 μM) + Ritonavir (10 μM)	3.4% ± 4.0%	2.2% ± 0.4%
Ribavirin (40 μM) + Ritonavir (20 μM)	1.9% ± 0.4%	3.6% ± 0.1%
Ribavirin (40 μM) + Ritonavir (35 μM)	4.6% ± 2.0%	9.8% ± 2.1%

* LDH release was determined in the culture supernatants from the final day of cultivation (60 days after the start of drug treatment) in order to examine the cytotoxicity caused by the drug treatment. Data represent the mean ± SD of triplicate wells.

## Data Availability

All data are presented in the manuscript.
